# Combining the differentiating effect of panobinostat with the apoptotic effect of arsenic trioxide leads to significant survival benefit in a model of t(8;21) acute myeloid leukemia

**DOI:** 10.1186/s13148-014-0034-4

**Published:** 2015-01-22

**Authors:** Jessica M Salmon, Michael Bots, Eva Vidacs, Kym L Stanley, Peter Atadja, Johannes Zuber, Ricky W Johnstone

**Affiliations:** Cancer Therapeutics Program, Peter MacCallum Cancer Centre, St. Andrews Place, East Melbourne, 3002 VIC Australia; Laboratory of Clinical Chemistry, Academic Medical Center, University of Amsterdam, Meibergdreef 9, 1105 AZ Amsterdam, The Netherlands; China Novartis Institutes for Biomedical Research, No. 2 BoYun Road, Pudong, Shanghai 201203 China; Research Institute of Molecular Pathology (IMP), Dr. Bohr-Gasse 7, A-1030 Vienna, Austria; The Sir Peter MacCallum Department of Oncology, University of Melbourne, Parkville, 3010 VIC Australia

**Keywords:** AML, Histone deacetylase inhibitor, Arsenic trioxide, Differentiation, Apoptosis, Therapy

## Abstract

**Background:**

One of the most frequently found abnormalities in acute myeloid leukemia (AML) is the t(8;21)(q22;q22) translocation, which is seen in around 15% of patients. This translocation results in the production of the AML1/ETO (A/E) fusion protein and commonly involves cooperating activating mutations of *RAS. AE9a* encodes a C-terminally truncated A/E protein of 575 amino acids that retains the ability to recruit histone deacetylases (HDACs). Expression of *AE9a* leads to rapid development of leukemia in experimental mouse systems. We have recently shown that treatment of mice bearing *A/E9a;Nras*^G12D^ tumors with the histone deacetylase inhibitor (HDACi) panobinostat leads to degradation of the A/E9a fusion protein, cell cycle arrest, differentiation of AML blasts into mature granulocytes and prolonged survival. Herein, we sought to enhance this therapeutic effect.

**Findings:**

Combined treatment of mice bearing *A/E9a;Nras*^G12D^ leukemias with panobinostat and arsenic trioxide (ATO) resulted in a significant survival advantage compared to mice treated with either agent alone. Moreover, some of the mice treated with the panobinostat/ATO combination showed complete tumor responses and remained in remission for over 220 days. Panobinostat caused differentiation of *A/E9a;Nras*^G12D^ cells while ATO induced apoptosis of the leukemic cells, an effect that was enhanced following co-treatment with panobinostat.

**Conclusions:**

Our results indicate that leukemic blast differentiation mediated by panobinostat combined with induction of apoptosis by ATO could be therapeutically beneficial and should be considered for patients with t(8;21) AML.

**Electronic supplementary material:**

The online version of this article (doi:10.1186/s13148-014-0034-4) contains supplementary material, which is available to authorized users.

## Findings

One of the most frequently found abnormalities in acute myeloid leukemia (AML) is the t(8;21)(q22;q22) translocation, which is seen in around 15% of patients [1]. The eight twenty-one (ETO) portion of the AML1/ETO (A/E) fusion protein recruits histone deacetylases (HDACs) providing a molecular rationale for using HDAC inhibitors (HDACi) as a therapeutic strategy. We have generated a transplantable model of mouse AML driven by concomitant expression of the AML1/ETO9a splice variant of *A/E* (*AE9a)* and *Nras*^G12D^ that recapitulates the genetics and pathology of human t(8;21) AML [2]. Treatment of mice bearing *A/E9a;Nras*^G12D^ tumors with the HDACi panobinostat leads to degradation of the A/E9a fusion protein, cell cycle arrest, differentiation of AML blasts into mature granulocytes, and prolonged survival [3].

### A combination of panobinostat and arsenic trioxide significantly increases the survival of mice transplanted with *A/E9a;Nras*^G12D^ tumors

**Figure 1:**
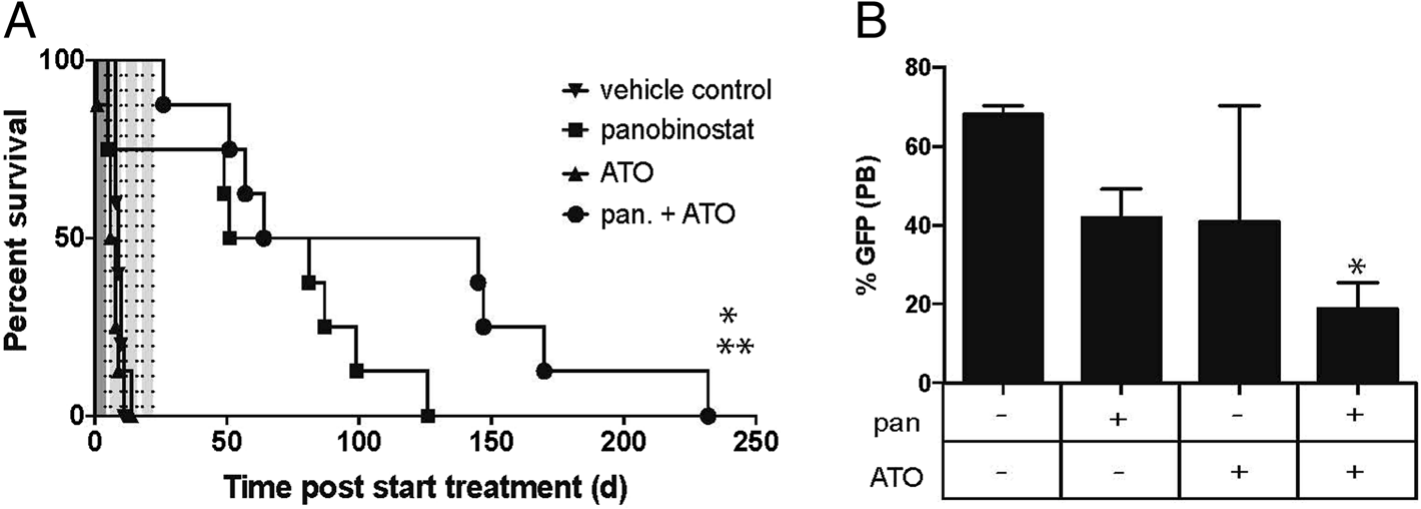
**A combination of the HDACi panobinostat and arsenic trioxide (ATO) demonstrates greater therapeutic efficacy against** ***A/E9a;Nras*** **-driven tumors. (A)** Kaplan-Meier survival curves of mice bearing *A/E9a;Nras* ^G12D^-driven leukemias treated with either vehicle (downward triangle), panobinostat (square), ATO (upward triangle) or a combination of panobinostat (pan) and ATO (circle). (n = 6 mice/group. Median survival benefit of panobinostat + ATO combination over panobinostat alone is 38.5 days; *P <0.05. and 95.5 days over vehicle alone; **P <0.001). Dotted lines indicate treatment days (5 days/ week; Monday through Fri) with the darker shaded area indicating the first week with a high dose of panobinostat (25 mg/kg) and the lighter shaded areas indicating the following three weeks with a lower dose of panobinostat (15 mg/kg). **(B)** Flow cytometry analysis of GFP-positive cells in the peripheral blood of mice treated for 5 days as indicated. (n = 3 to 6 mice per treatment group. Data are represented as the mean plus the standard error of the mean (SEM). *P <0.02).

Given our recent studies demonstrating that treatment of mice bearing *A/E9a;Nras*^G12D^ tumors with panobinostat resulted in significant therapeutic efficacy [3], we examined the effect of combining panobinostat with ATO in this model. The C57Bl/6 mice transplanted with *A/E9a;Nras*^G12D^ leukemia cells were treated with either vehicle, panobinostat alone, ATO alone, or the combination of panobinostat and ATO (Figure 1A). The treatment schedule involved an initial week of a high dose of panobinostat (darker shaded area) followed by three weeks of a lower dose of panobinostat combined with ATO (lighter shaded area). As previously reported [3], we observed a clear survival benefit following treatment with panobinostat alone (median survival 66 days). Strikingly, treatment with a combination of panobinostat and ATO extended this survival window (median survival was 105 days) with some individual mice showing no detectable tumor cells in the peripheral blood several months after cessation of therapy (not shown). Treatment with ATO alone resulted in no significant survival advantage compared to vehicle-treated mice (7 days versus 9 days, respectively). To examine the acute effect of the ATO and panobinostat combination, we analyzed tumor burden following 5 days of therapy. The percentage of GFP-positive cells in the peripheral blood was reduced following single-agent treatment with panobinostat or ATO alone (Figure 1B) with a more substantial reduction in tumor burden demonstrated following combination treatment with panobinostat and ATO (Figure 1B). These results demonstrate that while ATO alone has a marginal effect on *A/E9a;Nras*^G12D^ tumor cells, it has no effect on overall survival. However, when combined with panobinostat, ATO confers a significant survival advantage, superior to that of panobinostat alone.

### Arsenic trioxide causes leukemia cell death

**Figure 2:**
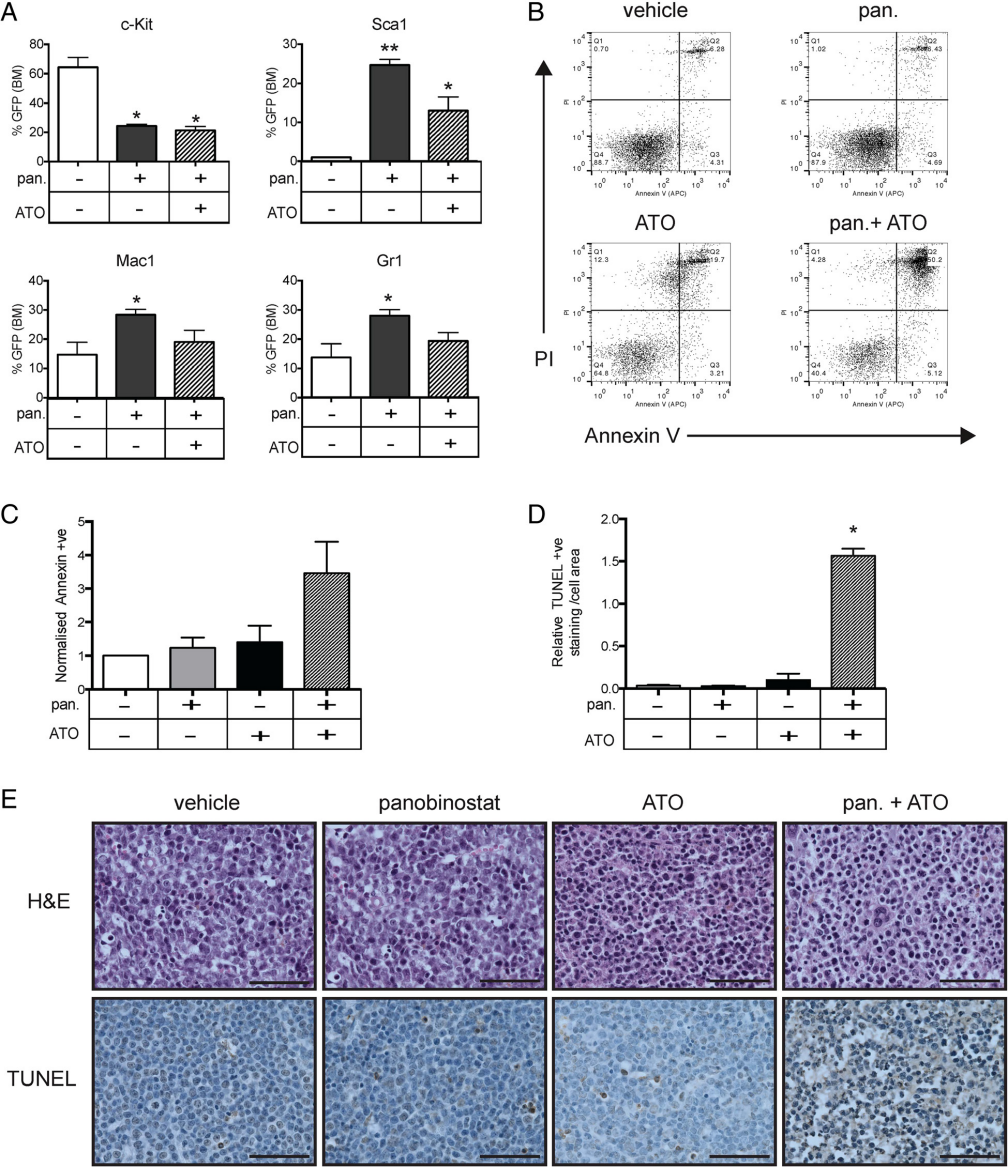
**The combined effects of panobinostat and arsenic trioxide (ATO) include the differentiation of** ***A/E9a;Nras*** ^**G12D**^-**leukemic blasts by panobinostat and induction of apoptosis by ATO. (A)** Flow cytometry analysis of the cell surface expression of c-Kit; Sca1; Mac1 and Gr1 of GFP-positive tumor cells in the bone marrow of *A/E9a;Nras* ^G12D^ tumor- bearing mice treated for 5 days with either panobinostat (pan; 25 mg/kg) or panobinostat combined with ATO (2.5 mg/kg). (n = 3; data are expressed as mean plus SEM; *P <0.05, **P <0.001) **(B)** Representative dot plots of AnnexinV-PI staining of tumor cells isolated from the bone marrow of A/E9a; Nras^G12D^ tumor-bearing mice treated for 4 hours with either panobinostat (25 mg/kg) or ATO (2.5 mg/kg) or a combination. Numbers given are the percentage of total cell population. **(C)** Normalized expression of AnnexinV on tumor cells treated with vehicle, panobinostat, ATO or a combination (n = 3; data are expressed as mean plus SEM). **(D)** Quantification of terminal deoxynucleotidyltransferase-mediated dUTP nick end labeling (TUNEL) positivity as a proportion of total cell area (n = 3; *P <0.001) **(E)** Representative images of hematoxylin-eosin staining (H&E; upper panels) and analysis of apoptotic cells by TUNEL staining, and counterstained with hematoxylin (lower panels). Staining was performed on de-calcified femurs isolated from *A/E9a;Nras* ^G12D^ tumor-bearing mice treated for 4 hours as indicated. Imaging was performed using 60x objective (scale bars = 50 μm).

We next sought to investigate how the panobinostat and ATO combination leads to increased survival benefit. Because panobinostat leads to terminal differentiation of *A/E9a;Nras*^G12D^ cells [3], we began by examining the effect on tumor cell differentiation caused by panobinostat with or without the addition of ATO. Mice bearing *A/E9a;Nras*^G12D^ tumors were treated with panobinostat with and without ATO, and the differentiation of tumor cells was examined at weekly intervals using flow cytometry to detect cell surface expression of myeloid markers c-Kit, Sca1, Mac1 and Gr1. A reduction of c-Kit and increased expression of Sca1, Mac1 and Gr1, indicative of terminal myeloid differentiation, was observed on *A/E9a;Nras*^G12D^ cells harvested from panobinostat-treated mice. Even after 3 weeks of therapy, the differentiation of tumor cells mediated by panobinostat was not enhanced following combination treatment (Figure 2A). These data indicate that ATO does not augment the differentiating effect of panobinostat despite further reducing tumor burden and enhancing survival of tumor-bearing mice.

We then analyzed the induction of tumor cell apoptosis following ATO treatment of mice transplanted with *A/E9a;Nras*^G12D^ cells. As we have previously demonstrated [3], panobinostat did not induce substantial tumor cell apoptosis 4 hrs after treatment, whereas combined treatment with panobinostat and ATO resulted in an increase in apoptotic *A/E9a;Nras*^G12D^ cells (Figure 2B and C). The apoptotic effect of the combination of panobinostat and ATO was more striking *in vivo* as assessed by TUNEL assay. Bone marrow sections from tumor-bearing mice treated with the panobinostat and ATO combination demonstrated high TUNEL-positivity after 4 hours compared to vehicle or the single agents alone (Figure 2D and E). Thus, the enhanced therapeutic effect of panobinostat and ATO is likely due to the combined effects of differentiation induced by panobinostat and apoptosis induced by ATO.

### Degradation of promyelocytic leukemia protein occurs in response to arsenic trioxide in acute myeloid leukemia tumors

**Figure 3:**
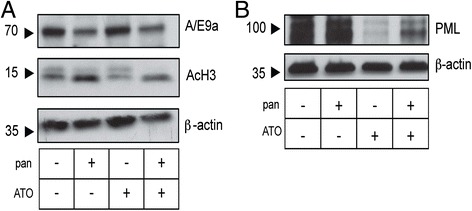
**Arsenic trioxide (ATO) does not enhance degradation of the Ae9a fusion protein or the cell cycle arrest induced by panobinostat but does lead to degradation of promyelocytic leukemia (PML) protein in** ***A/E9a;Nras*** ^**G12D**^ **tumor cells. (A)** Immunoblotting of whole cell lysates prepared from *A/E9a;Nras* ^G12D^ tumor cells treated *in vitro* with either panobinostat (pan; 16 μM), ATO (2.5 μM) or a combination, for 24 hours using antibodies against A/E9a or acetylated histone H3 (AcH3), with β-actin serving as a loading control. **(B)** Immunoblotting of whole cell lysates prepared from *A/E9a;Nras* ^G12D^ tumor cells treated *in vitro* with either panobinostat (16 μM), ATO (2.5 μM) or the combination, for 24 hours using antibodies against PML with β-actin serving as a loading control. The results shown are representative of three experiments.

Panobinostat causes degradation of A/E and A/E9a that precedes induction of tumor cell differentiation [3], and we sought to determine the effect of the panobinostat and ATO combination on expression of A/E9a (Figure 3A). Treatment with panobinostat but without ATO resulted in the degradation of A/E9a, and this depletion of the oncogenic fusion protein was not enhanced following addition of ATO (Figure 3A). Immunoblot for acetylated Histone H3 was also performed to confirm the HDAC inhibitory activity of panobinostat, and we observed no change in histone acetylation following ATO treatment (Figure 3A).

To further detect changes associated with ATO that correlate with therapeutic efficacy in mice bearing *A/E9a;Nras*^G12D^ tumors, we investigated the levels of PML protein levels by immunoblot. ATO binding induces PML oligomerization, which increases its interaction with the small ubiquitin-like modifier (SUMO)-conjugating enzyme 9, resulting in enhanced SUMOylation and degradation of PML [4]. No change in PML expression was observed in *A/E9a;Nras* tumor cells treated *in vitro* for 24 hours with either vehicle (DMSO) or panobinostat (Figure 3B). In contrast, treatment with ATO in the presence or absence of panobinostat resulted in a substantial decrease in PML expression (Figure 3B).

## Discussion

The successful introduction of ATO in combination with retinoids for the treatment of t(15;17) acute promyelocytic leukemia (APL) has resulted in cure rates of up to 90% in both newly diagnosed and relapsed APL patients with few side effects [5,6]. The mechanisms of ATO-mediated tumor cell responses include inactivation of key proliferation and survival pathways including the hedgehog, MAP kinase, NfκB and PI3K/AKT signaling pathways [5]. In addition, increased oxidative stress, downregulation of Bcl2, and degradation of PML nuclear bodies have also been implicated [5-9]. These multiple mechanisms of action of ATO suggest that it may have pro-apoptotic activity in hematological malignancies other than APL and chronic myeloid leukemia (CML) and that its use in combination with other targeted therapies may also be beneficial. In the present study, we examined the effect of the combination of ATO with the HDAC-inhibitor panobinostat using a pre-clinical mouse model of t(8;21) AML. Our results indicate that the additive effects of leukemic blast differentiation mediated by panobinostat with induction of apoptosis by ATO could be therapeutically beneficial.

Although HDAC-inhibitors have been minimally successful in the clinic when used as single agents, it is likely that they will be best utilized when combined with other anti-cancer agents. At the onset of this investigation, we performed toxicity studies on mice not bearing tumors to determine the maximum tolerated dose (MTD) of ATO as a single agent or in a combination. Upon establishing our dosing schedule, we observed an initial but not significant weight loss and no significant additional hematological toxicity (see Additional file 1: Figure S1A). Importantly, thrombocytopenia, which is the major dose-limiting toxicity associated with HDACi, in mice receiving the combination of panobinostat and ATO, was similar during the course of therapy to that of mice receiving panobinostat alone (see Additional file 1: Figure S1B).

Contrary to our observations, the combination of HDAC-inhibitors and ATO had previously been reported to be antagonistic in some leukemic cell lines [10]. Using our pre-clinical model of t(8;21) AML we demonstrated striking combined therapeutic efficacy using panobinostat and ATO. Panobinostat induces rapid degradation of A/E9a and A/E that precedes tumor cell differentiation [3]. Treatment with ATO alone had no effect on the expression of A/E9a and did not alter the panobinostat response when used in combination.

While much of the focus has centered on the degradation of PML and PML-RARα in APL in response to arsenic [11], there are numerous studies reporting the pro-apoptotic actions of ATO via PML degradation in non-APL cells [12]. Cells ectopically expressing A/E have been demonstrated to have increased PML bodies [13] and altered nuclear structure [14] and thus may be sensitized to PML degradation. It remains unclear why ATO as a single agent had no effect on the survival of mice bearing *A/E9a;Nras*^G12D^ leukemias as we did observe degradation of PML and apoptosis with the use of ATO as a single agent. In addition, we observed that the combination of panobinostat and ATO actually resulted in a decrease in PML degradation. PML is known to interact with HDACs [15,16], in particular, HDAC7 is required for the SUMOylation of PML which enhances its degradation [17]. It is possible that inhibition of HDAC activity leads to the partial rescue of PML degradation caused by ATO. Regardless, we demonstrate that combining panobinostat with the apoptosis-inducing agent ATO in mice bearing A/E9a/Nras^G12D^ tumors resulted in a significant survival advantage compared to tumor-bearing mice treated with single agents alone.

We propose that patients with t(8;21) AML may benefit from this type of targeted combination therapy. Indeed, a similar strategy has been extraordinarily successful in improving the treatment for acute promyelocytic leukemia resulting from the t(15;17) translocation. In that subtype of AML, treatment with all-*trans*-retinoic acid (ATRA) and ATO results in degradation of the PML-RARα fusion oncogene, differentiation of tumor cells, and apoptosis in leukemic blasts. The similarities between our results using panobinostat in combination with ATO in mice bearing *A/E9a;Nras*^G12D^ tumors and the effects of retinoids and ATO in models of PML-RARα-driven APL indicate that, as differentiation therapy combined with ATO has been so successful in curing patients with PML, the combination of panobinostat and ATO for relapsed t(8;21) patients may have similar success.

## Methods

### Mice and reagents

All reported research involving the use of animals was approved by the Animal Experimentation Ethics Committee (AEEC) at the Peter MacCallum Cancer Centre (ref. E472). C57BL/6 mice were purchased from the Walter and Eliza Hall Institute of Medical Research. Panobinostat was provided by Novartis and prepared as a 2 mg/mL solution in 5% dextrose/dH_2_O (D5W). Arsenic trioxide (Phenasen™) was obtained from the Peter MacCallum Cancer Centre and further diluted in phosphate-buffered saline to a 0.5 mg/mL solution.

### Mouse transplants

Mouse tumors expressing MSCV-AML1/ETO9a-IRES-GFP (A/E9a) and MSCV-luciferase-IRES-Nras^G12D^ (*A/E9a;Nras*^G12D^) were generated as previously described [3]. At the terminal disease stage, mice were euthanized and leukemia cells were isolated from the bone marrow and spleen. Single-cell suspensions were prepared, and cells were cryopreserved in fetal calf serum (FCS)/10% dimethylsulfoxide (DMSO). Total white blood cell (WBC) counts were obtained using the CELL-DYN Sapphire Hematology System (Abbott Laboratories, Lane Cove, NSW, Australia).

To perform therapeutic studies, *A/E9a;Nras*^G12D^ leukemia cells (1 × 10^6^) were then serially transplanted into sublethally irradiated mice by intravenous injection. Treatment was initiated once the leukemic burden reached between 5% and 20% GFP-positive cells in peripheral blood. Mice were treated daily for 5 consecutive days per week via intraperitoneal injection over 4 weeks. Treatment cohorts were either (1) ATO (2.5 mg/kg); (2) panobinostat (25 mg/kg) for 1 week followed by 15 mg/kg for 3 weeks; (3) panobinostat (25 mg/kg) for 1 week followed by panobinostat (15 mg/kg) combined with ATO (2.5 mg/kg); or (4) control mice receiving an equivalent volume of vehicle.

### Cell culture conditions

*A/E9a;Nras*^G12D^ leukemia cells were cultured *ex vivo* at 37°C under 5% CO_2_ in RPMI-1640 (Gibco, Grand Island, NY, USA) containing 20% FCS supplemented with 10 ng/mL IL-3, 10 ng/mL IL-6 and 50 ng/mL SCF (all Peprotech, Rocky Hill, NJ, USA). Cells were treated with panobinostat (16 nM) and/or ATO (2.5 μM) for 24 hours or the equivalent volume of vehicle.

### Western blot analysis

Whole cell lysates were separated by sodium dodecyl sulfate polyacrylamide gel electrophoresis and transferred onto polyvinylidene difluoride membranes (Millipore, Darmstadt, Germany). Membranes were blocked with 5% nonfat milk in Tris-buffered saline/0.1% Tween-20 at room temperature and incubated overnight with antibodies against AML1 (4336; Cell Signaling Technology, Danvers, MA, USA), acetyl-histone H3 (06–599; Millipore), PML (05–718; Millipore), or β-actin (A2228; Sigma-Aldrich, St Louis, MO, USA) at 4°C. Membranes were developed using appropriate horseradish peroxidase–coupled secondary antibodies (Dako, Glostrup, Denmark) and enhanced chemiluminescence (GE Healthcare, Little Chalfont, UK).

### Differentiation markers

Cell suspensions were incubated in red cell lysis buffer (150 mM NH_4_Cl, 10 mM KHCO_3_, 0.1 mM EDTA) and washed twice in fluorescence-activated cell sorter (FACS) staining buffer (phosphate-buffered saline supplemented with 2% FCS and 0.02% NaN_3_). Cells were pre-incubated with blocking anti-CD16/CD32 (2.4G2) and stained on ice with antibodies specific for c-Kit (CD117), Sca1 (Ly6A/E), Mac-1 (CD11b) and Gr1 (Ly6G; all BD Biosciences, San Jose, USA) in FACS staining buffer for 30 minutes. Data were collected on a FACSCanto II flow cytometer (BD Biosciences) and analyzed using FlowJo software (Tree Star, http://www.flowjo.com).

### Cell death assays

Terminal deoxynucleotidyltransferase-mediated dUTP nick end labeling (TUNEL) staining was performed using the Apoptag Peroxidase In Situ Apoptosis Detection Kit (Millipore). Sections were counterstained using hematoxylin, and images were recorded with a Zeiss microscope and a × 60 lens. Quantitation of positive TUNEL staining was performed using MetaMorph™ Microscopy Automation & Image Analysis Software (http://www.moleculardevices.com/metamorph).

Cells were isolated from the femurs of treated mice and stained with APC-conjugated AnnexinV (BD Biosciences) and 1 μg/ml propidium iodide in AnnexinV binding buffer (10 mM HEPES/NaOH [pH 7.4], 140 mM NaCl, 5 mM CaCl_2_x2H_2_O) to detect dead or dying cells by flow cytometry. Data were collected on a FACSCanto II flow cytometer (BD Biosciences) and analyzed using FlowJo software (Tree Star).

### Statistical analysis

Kaplan-Meier survival curves were created, and survival of mice (using a log-rank test) was statistically analyzed with GraphPad Prism software (http://www.graphpad.com/scientific-software/prism/). All other statistical analyses (using a 2-tailed unpaired Student *t* test) were also performed with this software.

## Additional file

Additional file 1: Figure S1. Combination therapy of panobinostat and arsenic trioxide (ATO) has no significant toxicity over single agents alone. (A) Mice were treated with either ATO alone or with a combination of ATO and panobinostat. Weight of treated mice was monitored daily throughout the course of therapy. Data are mean plus SEM. n = 6 mice per treatment group. (B) Platelet counts from mice treated with either vehicle, panobinostat, or a combination of panobinostat and ATO. Peripheral blood was taken to monitor thrombocytopenia throughout the therapy at weekly intervals. Data are mean plus SEM, n = 3 mice per treatment group.

## Abbreviations

AML: Acute myeloid leukemia
APL: Acute promyelocytic leukemia
ATO: Arsenic trioxide
CML: Chronic myeloid leukemia
ETO: Eight twenty-one
HDAC: Histone deacetylase
PML: Promyelocytic leukemia
TUNEL: Terminal deoxynucleotidyltransferase-mediated dUTP nick end labeling
