# Correction to: Combining the differentiating effect of panobinostat with the apoptotic effect of arsenic trioxide leads to significant survival benefit in a model of t(8;21) acute myeloid leukemia

**DOI:** 10.1186/s13148-020-00964-9

**Published:** 2020-11-18

**Authors:** Jessica M. Salmon, Michael Bots, Eva Vidacs, Kym L. Stanley, Peter Atadja, Johannes Zuber, Ricky W. Johnstone

**Affiliations:** 1 Cancer Therapeutics Program, Peter MacCallum Cancer Centre, St. Andrews Place, East Melbourne, VIC 3002 Australia; 2 Laboratory of Clinical Chemistry, Academic Medical Center, University of Amsterdam, Meibergdreef 9, 1105 AZ Amsterdam, The Netherlands; 3 China Novartis Institutes for Biomedical Research, No. 2 BoYun Road, Pudong, Shanghai, 201203 China; 4 Research Institute of Molecular Pathology (IMP), Dr. Bohr-Gasse 7, A-1030 Vienna, Austria; 5 The Sir Peter MacCallum Department of Oncology, University of Melbourne, Parkville, VIC 3010 Australia

## Correction to: Clinical Epigenetics (2015) 7:2, https://doi.org/10.1186/s13148-014-0034-4

Following publication of the original article [1], the authors identified an error in Fig. 2e. The correct Fig. 2 is given below.

**Fig. 2:**
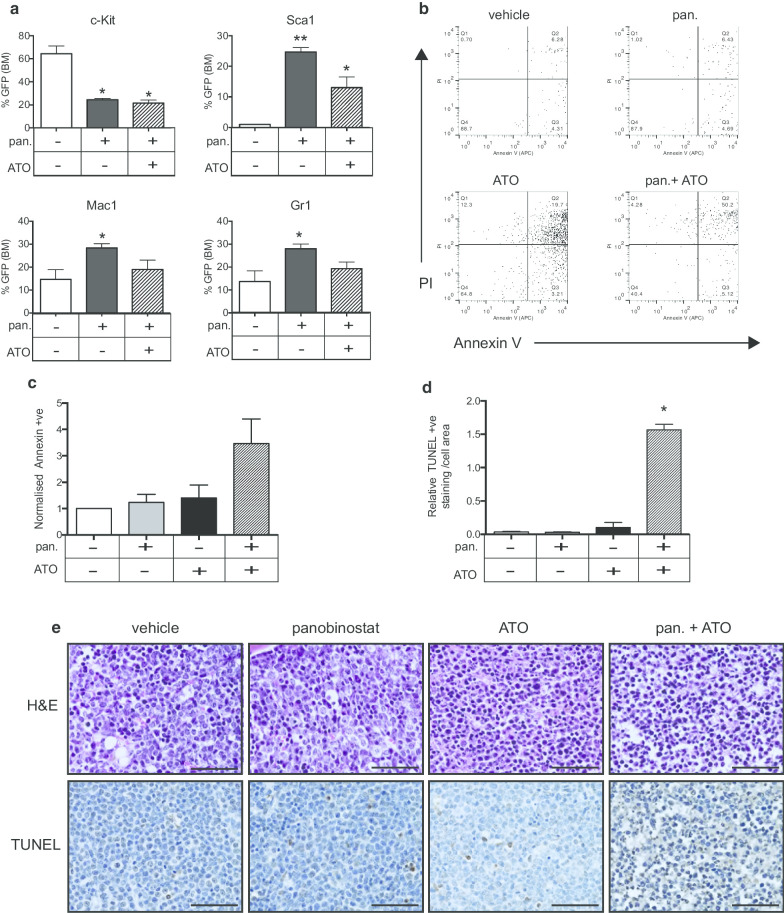
The combined effects of panobinostat and arsenic trioxide (ATO) include the differentiation of *A/E9a;Nras* ^G12D^-leukemic blasts by panobinostat and induction of apoptosis by ATO.** (A)** Flow cytometry analysis of the cell surface expression of c-Kit; Sca1; Mac1 and Gr1 of GFP-positive tumor cells in the bone marrow of *A/E9a;Nras* ^G12D^ tumor-bearing mice treated for 5 days with either panobinostat (pan; 25 mg/kg) or panobinostat combined with ATO (2.5 mg/kg). (n = 3; data are expressed as mean plus SEM; *P <0.05, **P <0.001) **(B)** Representative dot plots of AnnexinV-PI staining of tumor cells isolated from the bone marrow of A/E9a; Nras^G12D^ tumor-bearing mice treated for 4 hours with either panobinostat (25 mg/kg) or ATO (2.5 mg/kg) or a combination. Numbers given are the percentage of total cell population. **(C)** Normalized expression of AnnexinV on tumor cells treated with vehicle, panobinostat, ATO or a combination (n = 3; data are expressed as mean plus SEM). **(D)** Quantification of terminal deoxynucleotidyltransferase-mediated dUTP nick end labeling (TUNEL) positivity as a proportion of total cell area (n = 3; *P <0.001) **(E)** Representative images of hematoxylin-eosin staining (H&E; upper panels) and analysis of apoptotic cells by TUNEL staining and counterstained with hematoxylin (lower panels). Staining was performed on de-calcified femurs isolated from *A/E9a;Nras* ^G12D^ tumor-bearing mice treated for 4 hours as indicated. Imaging was performed using 60x objective (scale bars = 50 μm).

