# Supplementary figures and images for: Combining the differentiating effect of panobinostat with the apoptotic effect of arsenic trioxide leads to significant survival benefit in a model of t(8;21) acute myeloid leukemia
Source: Clin Epigenetics. 2015 Jan 22;7(1):2. doi: 10.1186/s13148-014-0034-4 (PMC4308003; doi:10.1186/s13148-014-0034-4)

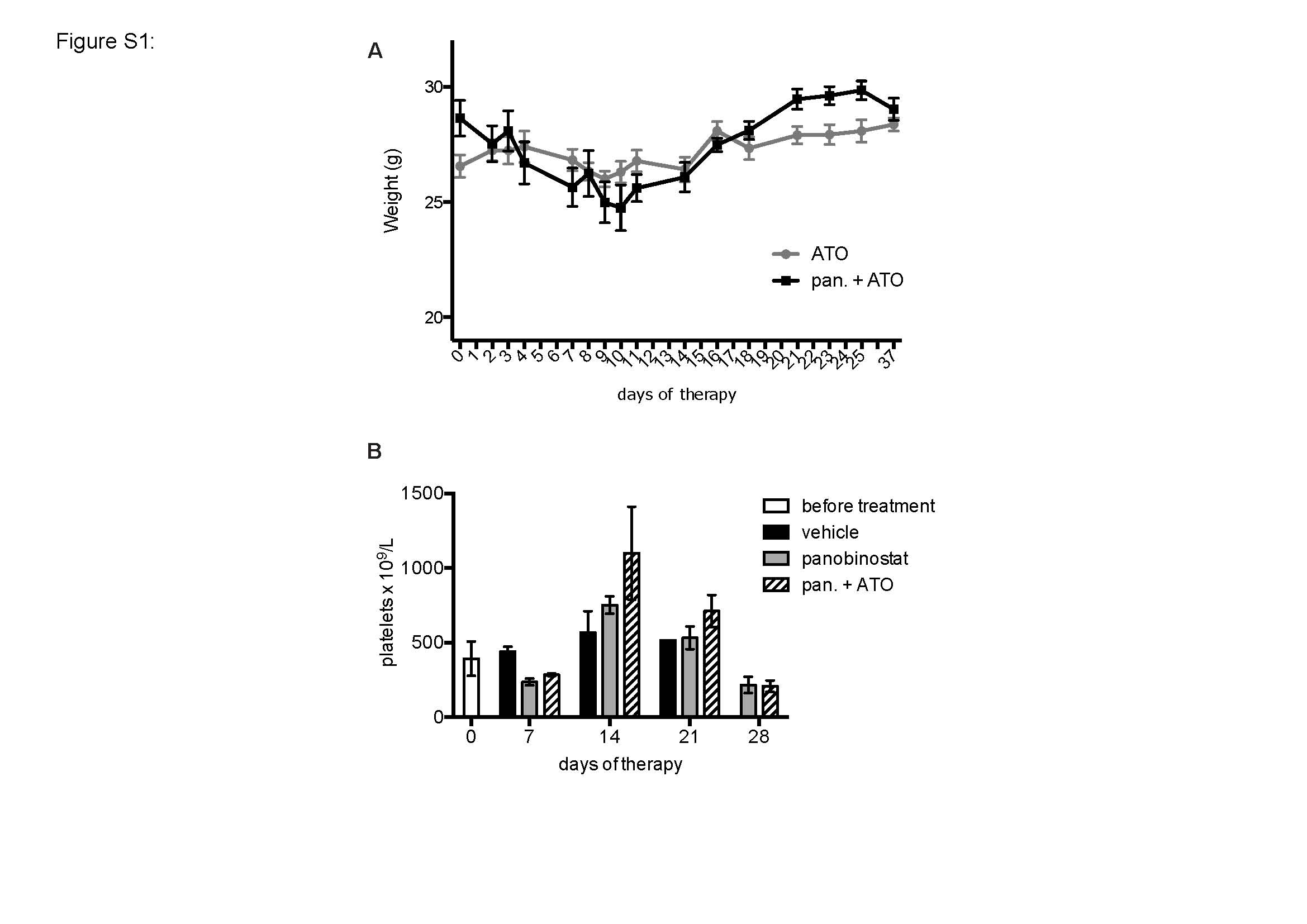

Supplement: Additional file 1: Figure S1. — Combination therapy of panobinostat and arsenic trioxide (ATO) has no significant toxicity over single agents alone. (A) Mice were treated with either ATO alone or with a combination of ATO and panobinostat. Weight of treated mice was monitored daily throughout the course of therapy. Data are mean plus SEM. n = 6 mice per treatment group. (B) Platelet counts from mice treated with either vehicle, panobinostat, or a combination of panobinostat and ATO. Peripheral blood was taken to monitor thrombocytopenia throughout the therapy at weekly intervals. Data are mean plus SEM, n = 3 mice per treatment group. [file 13148_2014_34_MOESM1_ESM.jpeg]
